# Current Concepts and Future Developments of Corneal Cross-Linking

**DOI:** 10.1155/2015/302983

**Published:** 2015-03-24

**Authors:** Suphi Taneri, Elias Jarade, John A. Kanellopoulos, David Muller

**Affiliations:** ^1^Center for Refractive Surgery, Eye Department, St. Francis Hospital, 48145 Münster, Germany; ^2^Cornea and Refractive Surgery Department, International Medical Center, Dubai, UAE; ^3^LaserVision Eye Institute, Athens, Greece; ^4^New York University School of Medicine, New York, NY, USA; ^5^Avedro, Inc., Waltham, MA, USA

The introduction of corneal cross-linking (CXL) has changed the landscape of treatment and management of keratoconus and ectasia after refractive surgery. Previously, treatment options provided only temporary visual rehabilitation without limiting disease progression. Keratoconus and ectasia resulted in significant vision-related reduction in quality of life and a substantial lifetime economic burden, with up to 20% of keratoconus cases resulting in eventual corneal transplantation [1]. CXL was introduced as the first therapeutic option for keratoconus aimed at stiffening the cornea in order to treat the underlying stromal instability. From the time that the first reports of the clinical application of cross-linking in the cornea were published in 2003 [2], CXL has rapidly been adopted as a standard therapy for treatment of progressive keratoconus in much of the world [3–5].

The potential for early intervention with corneal cross-linking* before* visual function has been compromised has resulted in a shift in the way we think about corneal biomechanics and has reawakened interest in the early diagnosis of ectasia. In this issue, J. Steinberg et al. report two new parameters to detect biomechanical changes in the keratoconic cornea after CXL using in vivo corneal visualization Scheimpflug technology. Research in the area of corneal biomechanics has the potential to enable earlier diagnosis of patients in need of CXL and better analysis of the effects of the procedure.

While many questions remain unanswered, the understanding of the photochemical mechanisms that result in the formation of cross-links in the cornea has grown exponentially in the last decade. Improved scientific understanding of the mechanisms of CXL has driven clinical research aimed at optimizing CXL for better efficiency and efficacy [6]. In this issue, A. C. da Paz et al. present a critical review of known and as yet undetermined effects of CXL on corneal structure, biomechanics, and functional aspects. J. Antoun et al. examine patient characteristics that contribute to cross-linking failure to stabilize keratometry and examine the effect of repeat treatments on eyes that have continued to progress after primary CXL.

The remaining contributions to the special issue explore modifications to the conventional protocol that incorporate new technology, such as transepithelial riboflavin formulations designed to improve patient comfort (S. Taneri et al.) and higher irradiance and pulsed UVA delivery aimed at improving procedure speed and photon efficiency (C. Mazzotta et al.). CXL combination procedures targeted at maximizing visual outcomes through the addition of simultaneous intracorneal ring implantation (P. Studeny et al.) or subsequent phakic toric implantable collamer lens insertion (R. Antonios et al.) are also discussed.

Together, the papers in this special issue describe the next generation of corneal cross-linking. The contributors explore the potential to maximize CXL efficacy, provide equivalent or greater treatment effect in shorter total treatment times, reduce patient discomfort and speed visual recovery, and offer both stabilization and functional visual improvement through CXL combination procedures.

*Suphi Taneri* *Suphi Taneri* *Elias Jarade* *Elias Jarade* *John A. Kanellopoulos* *John A. Kanellopoulos* *David Muller* *David Muller*
